# *Bartonella quintana* Endocarditis in Dogs

**DOI:** 10.3201/eid1212.060724

**Published:** 2006-12

**Authors:** Patrick Kelly, Jean-Marc Rolain, Ricardo Maggi, Sushama Sontakke, Bruce Keene, Stuart Hunter, Hubert Lepidi, Kyle T. Breitschwerdt, Edward B. Breitschwerdt, Didier Raoult

**Affiliations:** * Ross University, Basseterre, St Kitts, West Indies;; † Unité des Rickettsies, Marseille, France;; ‡ North Carolina State University, Raleigh, North Carolina, USA;; § Massey University, Palmerston North, New Zealand

**Keywords:** Bartonella quintana, endocarditis, canine, research

## Abstract

We provide the first evidence that *Bartonella quintana* can infect dogs and cause typical signs of endocarditis. Using PCR and sequencing, we identified *B. quintana* in the blood of a dog from the United States with aortic valve endocarditis and probably also in the mitral valve of a dog from New Zealand with endocarditis.

## Abstract 2

TOC summary line: PCR and sequencing provide the first evidence that *B. quintana* can be pathogenic in dogs.

Bacterial endocarditis is an uncommon, often fatal, disease of dogs (*1*). Although a variety of bacteria can be isolated with routine blood cultures, Bartonella spp., gram-negative bacteria with fastidious growth requirements, are the most common etiologic agents (*1*). While B. vinsonii subsp. berkhoffii (*2*), B. clarridgeiae (*3*), and B. washoensis (*4*) cause endocarditis in dogs, B. henselae and B. quintana are the most common species that cause endocarditis in humans (*5*). We provide the first evidence that B. quintana can also infect dogs and cause endocarditis.

## Case 1

A 3-year-old castrated mixed breed dog was referred to the Veterinary Teaching Hospital of North Carolina State University on December 21, 1999, with lethargy, anorexia, fever, occasional cough, and lameness in the right rear leg of 8 days' duration. The dog was maintained mostly indoors but lived on a farm and was occasionally observed chasing wild animals or fighting with the other 5 dogs in the household. It also had frequent exposure to 2 pet cats. At the time of the dog's illness, all other pets in the household were considered healthy. Physical examination showed a grade 4/6, to-and-fro murmur and changes in the right rear leg compatible with vascular occlusion secondary to thromboembolism. Laboratory abnormalities included marked neutrophilia (43,000/μL, reference range 3,000–11,000/μL) and mild lymphocytosis, eosinophilia, hypoalbuminemia, and hyperglobulinemia. Thoracic radiographs showed mild left atrial enlargement and mild pulmonary interstitial infiltrates. Results of an electrocardiogram were normal, but echocardiography showed a large vegetative lesion on the aortic valve that caused stenosis and severe insufficiency.

After 1 week's treatment with oral amoxicillin-clavulanate, enrofloxacin, enalapril, atenolol, and subcutaneous heparin sodium, the lameness resolved, and the demeanor was normal. Oral aspirin was substituted for heparin, and medications continued for 6 months, at which time the owner reported that the dog was healthy. On reexamination, the murmur was softer (grade 3/6), but the aortic valve vegetation and insufficiency persisted with progressive left atrial and ventricular enlargement. Subsequently, atrial fibrillation developed, and the dog died from refractory congestive heart failure on September 25, 2002. A necropsy was not performed.

## Case 2

In November 2003, a 3-year-old castrated mixed breed dog weighing 48 kg was referred to the Veterinary Teaching Hospital of Massey University, Palmerston North, New Zealand, for evaluation of a heart murmur. The dog lived mainly outdoors and had a 1-week history of depression, fever (40.1°C), and swelling of the left tarsus, which resolved with administration of ampicillin and clavulanate. On examination, the dog was febrile (39.8°C) and had marked dyspnea with mild cyanosis. Crackles were heard on both sides of the chest, and a grade 4/6 pansystolic murmur was loudest over the mitral valve area. The dog had numerous fleas (Ctenocephalides felis). Laboratory abnormalities included mild nonregenerative anemia, mature neutrophilia (23,000/μL, reference range 3,600–11,500/μL), mild hypoalbuminemia, and mildly elevated urea and creatinine levels. The urine was concentrated (1.033) and contained large numbers of granular casts.

The heart appeared normal in thoracic radiographs, but the pulmonary vasculature was mildly enlarged, and a marked diffuse alveolar pattern occurred throughout the lungs. The heart appeared normal on echocardiography.

Despite symptomatic treatment with fluids, furosemide, and amoxicillin-clavulanate, the dog's condition deteriorated rapidly, and the animal was euthanized at the owner's request. Permission was obtained for postmortem examination.

## Materials and Methods

### Case 1

Routine blood and urine cultures were performed. Specialized blood cultures for Bartonella that used blood agar plates and liquid cell culture medium (*6*) were obtained.

A year after the dog died, frozen (-80°C) stored aliquots of whole blood (200 μL) and the culture-negative liquid cell-culture medium (1 mL) were thawed, and DNA was extracted with the QIAamp DNA Mini Kit (Qiagen Inc., Valencia, CA, USA). PCR was performed with primers that amplify portions of the α-Proteobacteria citrate synthase gene (gltA) (5´ CAT GCA GAY CAR GAR CAR AAT GCT TCT AC 3´ and 5´ ATW CCN GAA TAA AAR TCA ACA TTN GGR TAH A 3´) and the phage-associated gene (pap31) found in several Bartonella spp. (Pap31 1(s): 5´ GAC TTC TGT TAT CGC TTT GAT TT 3´ and Pap31 688 (as): 5´ CAC CAC CAG CAA MAT AAG GCA T 3´), as described previously (*7*). With both primer sets, products were amplified by using DNA from the whole blood and the liquid cell-culture medium. The amplicons were cloned with the pGEM-T Easy Vector System (Promega, Madison, WI, USA) and the sequences determined by Davis Sequencing, Inc. (Davis, CA, USA). Sequences obtained were compared with those in GenBank by using AlignX software (Vector NTI Suite 6.0, InforMax, Inc., Invitrogen Corp., Carlsbad, CA, USA).

### Case 2

Abnormal tissues found at postmortem examination were fixed in 10% formalin, embedded in paraffin, and sectioned and routinely stained with hematoxylin and eosin. Immunohistochemical testing was performed with rabbit anti–B. quintana antibody (1:1000) and hematoxylin counterstaining as described previously (*8*).

DNA was extracted from the formalin-fixed mitral valve with the QIAamp DNA Mini Kit (Qiagen GmbH, Hilden, Germany). PCR was performed with primers for gltA and the ITS fragment as described previously (*3**,**4*). Also, a 1-step LightCycler nested PCR was performed as previously described (*9*) with external and internal primers amplifying the fur gene (*10*). PCR products were purified with the QIAquick PCR Purification Kit (Qiagen) and sequenced with the dRhodamine Terminator Cycle Sequencing Ready Reaction Mix (Applied Biosystems, Foster City, CA, USA) and an ABI PRISM 310 DNA Sequencer (Applied Biosystems). Multiple alignments were made with the sequences obtained with the Clustal W software, version 1.81 (*11*).

## Results

### Case 1

Routine blood and urine cultures and specialized blood cultures for Bartonella were negative. Amplicons were obtained with primers for the gltA (422 bp; GenBank accession no. DQ383817) and the pap31 (526 bp; GenBank accession no. DQ383818). These sequences had 99% (gltA) and 99.8% (pap 31) homology with B. quintana Fuller (GenBank accession no. BQCSFULLR) and B. quintana strain Toulouse (GenBank accession no. BX897700), respectively.

### Case 2

On postmortem examination, severe congestion and edema of the lungs with blood-tinged pleural (250 mL) and pericardial (75 mL) effusion were evident. Although the heart was of normal size and shape, multiple soft, friable, irregular red masses, the largest measuring 10 mm in diameter, were firmly attached to 3 cusps of the mitral valve. The aortic valve was normal. Histologic sections of the mitral valve showed multifocal erosions of the endothelium that contained large masses of fibrin admixed with pockets of degenerate neutrophils. While bacteria were not seen in hematoxylin and eosin or gram-stained sections, Warthin-Starry staining showed multiple clusters of rod-shaped organisms within the masses of fibrin. The organisms were also seen by immunohistochemistry with the genus-reactive polyclonal rabbit anti–B. quintana antibody and hematoxylin counterstaining (Figure).

**Figure:**
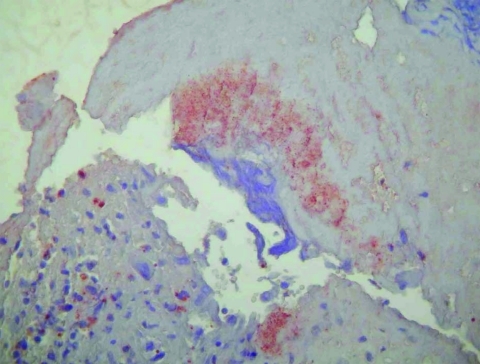
Immunohistochemical demonstration of bartonellae in the mitral valve with peroxidase-conjugated polyclonal rabbit anti–*Bartonella* sp. antibodies. The organisms stain dark orange against the hematoxylin counterstain; original magnification ×200.

The spleen, pancreas, and left kidney had multiple areas of infarction and hemorrhage with numerous intravascular fibrin thrombi. Warthin-Starry–stained sections showed numerous organisms, similar to those found in the valvular masses, within and surrounding many of the thrombosed blood vessels.

No product was obtained with primers for gltA and the ITS fragment. Nested PCR that used the fur primers, however, did provide a 202-bp amplicon (GenBank accession no. DQ666269) that had 99% homology with B. quintana strain Toulouse (GenBank accession no. BX897700) and B. koehlerae (GenBank accession no. DQ666271). It had 97% homology with B. clarridgeiae strain 94-F40 (GenBank accession no. DQ683729) and lower levels with sequences in GenBank of B. bacilliformis (GenBank accession no. AF388198) and other Bartonella spp. known to cause endocarditis in dogs and humans: B. elizabethae (GenBank accession no. DQ666270), B. henselae strain Houston-1 (GenBank accession no. BX897699), and B. vinsoni subsp. berkhoffi (GenBank accession no. DQ666272).

## Discussion

The diagnosis of canine bacterial endocarditis is usually based on appropriate clinical and echocardiographic findings or typical pathologic lesions (*1*). The abnormalities we found were similar to those reported in dogs with bacterial endocarditis and endocarditis due to Bartonella spp., namely murmur (89%), fever (72%), leukocytosis (78%), hypoalbuminemia (67%), thrombocytopenia (56%), elevated liver enzymes (56%), lameness (43%), azotemia (33%), respiratory abnormalities (28%), and weakness and collapse (17%) (*1*). One dog had clear echocardiographic evidence of endocarditis; the other had distinct lesions at necropsy not seen with echocardiography.

B. quintana was the most likely cause of endocarditis identified in our dogs. In the first dog, routine blood cultures were negative for other bacteria that cause endocarditis. PCR and sequencing, however, demonstrated DNA of B. quintana in the dog's blood at the time endocarditis was diagnosed. Although specialized blood cultures for Bartonella spp. were negative, these organisms have fastidious growth requirements, and blood cultures that use solid media have poor diagnostic sensitivity in both humans (*9*) and dogs (*1*).

The most useful techniques for detecting Bartonella endocarditis are immunohistochemical analysis of affected valves and PCR (*1**,**5**,**8*). In case 2, the dog had typical histologic lesions of endocarditis that contained large numbers of Bartonella organisms, as shown by Warthin-Starry staining and immunohistochemical analysis. When the sequences of the fur gene were compared with those of Bartonella spp. that are known to cause endocarditis in dogs and humans, the sequencing results showed the Bartonella that infected the dog had highest homology (99%) with B. quintana and B. koehlerae. We did not have control DNA to test for B. washoensis, which has been described as an agent of endocarditis in a dog (*4*) and myocarditis in a human (*12*), but we regarded infection with this organism as unlikely because it has only been identified in the United States. Although we know of no specific reports of B. quintana in New Zealand, the organism is ubiquitous (*13*) and is the most likely cause of the endocarditis in the dog we studied. We decided the organism was not B. koehlerae because it has not been reported in New Zealand or found in recent studies of its natural host (domestic cat) and vector (cat flea) in New Zealand (*14**–**16*). Although the organism causes endocarditis in humans (*17*), it does not appear to be pathogenic in cats, the natural host (*18*).

Our description of B. quintana causing disease in the dog is the first report of the organism's pathogenicity in vertebrates other than humans, the natural reservoirs of the organism. Also, our report adds to the growing evidence that B. quintana can infect species other than humans. In recent reports, B. quintana was identified in a cat euthanized for medical reasons not related to infectious diseases (*19*) and in an apparently healthy captive-bred cynomolgus monkey (Macaca fascicularis) (*20*). B. quintana was first described as the agent of trench fever in soldiers in World War I. The organism causes a variety of clinical signs, including endocarditis, which is seen most commonly in immunocompetent, homeless men with a history of alcohol abuse (*5*). Although the body louse is the traditional vector of B. quintana in humans, this parasite was not a likely source of infection for our dogs since it is host specific, and we found no evidence of infestation. Recent reports of B. quintana in persons with no history of body lice have suggested that other vectors may be involved. In France, a high percentages (17%) of C. felis contain DNA of B. quintana, which suggests that cat fleas might be vectors (*21*). Although the dog from New Zealand had fleas, B. quintana has not been identified in C. felis in the country (*15**,**16*). Another proposed vector is Ixodes pacificus (*22*), but this tick does not occur in North Carolina or New Zealand. Further, ticks are very rarely found on dogs in New Zealand, where PCR studies on the only prevalent species, Haemaphysalis longicornis, have been negative for Bartonella spp (*23*). The source of the B. quintana infections in the dogs we describe is unclear.

In summary, our study has shown B. quintana can infect dogs and cause endocarditis. Further studies are indicated to investigate the epidemiology of these infections.
